# Machine Learning Assisted Wearable Wireless Device for Sleep Apnea Syndrome Diagnosis

**DOI:** 10.3390/bios13040483

**Published:** 2023-04-17

**Authors:** Shaokui Wang, Weipeng Xuan, Ding Chen, Yexin Gu, Fuhai Liu, Jinkai Chen, Shudong Xia, Shurong Dong, Jikui Luo

**Affiliations:** 1 Ministry of Education Key Laboratory of RF Circuits and Systems, College of Electronics & Information Hangzhou Dianzi University, Hangzhou 310018, China; 2 The Fourth Affiliated Hospital Zhejiang University School of Medicine, Yiwu 322000, China; 3 Key Laboratory of Advanced Micro/Nano Electronic Devices & Smart Systems of Zhejiang, College of Information Science & Electronic Engineering, Zhejiang University, Hangzhou 310027, China

**Keywords:** sleep apnea syndrome, photoplethysmography (PPG), wearable device, machine learning

## Abstract

Sleep apnea syndrome (SAS) is a common but underdiagnosed health problem related to impaired quality of life and increased cardiovascular risk. In order to solve the problem of complicated and expensive operation procedures for clinical diagnosis of sleep apnea, here we propose a small and low-cost wearable apnea diagnostic system. The system uses a photoplethysmography (PPG) optical sensor to collect human pulse wave signals and blood oxygen saturation synchronously. Then multiscale entropy and random forest algorithms are used to process the PPG signal for analysis and diagnosis of sleep apnea. The SAS determination is based on the comprehensive diagnosis of the PPG signal and blood oxygen saturation signal, and the blood oxygen is used to exclude the error induced by non-pathological factors. The performance of the system is compared with the Compumedics Grael PSG (Polysomnography) sleep monitoring system. This simple diagnostic system provides a feasible technical solution for portable and low-cost screening and diagnosis of SAS patients with a high accuracy of over 85%.

## 1. Introduction

With the rapid progress of modern society, continuous improvement in the quality of people’s lives and living standards has gained more and more attention. Sleeping-associated diseases have become one of the most serious health problems due to the increased work-associated stress and the fast pace of modern society. Sleep apnea syndrome (SAS) is a representative of sleep-disordered breathing diseases that constantly threaten human health. When breathing stops for about ten seconds or more during sleep unintentionally, apnea occurs. Repeated nocturnal hypoxia and hypercapnia due to SAS can lead to complications such as hypertension, coronary heart disease, diabetes, cerebrovascular disease, traffic accidents, and even sudden death at night. According to statistics, the global prevalence of SAS is about 4% for men and 2% for women, and it increases with the age of the population. For example, the prevalence of SAS among seniors over 65 is 20% to 40% [1]. Therefore, early diagnosis and screening for sleep-disordered breathing diseases will be effective in preventing SAS.

For the diagnosis of sleep apnea, most medical institutions rely on the doctor’s personal experience and physical examination results. These contain artifacts and human errors. There are also some medical tools, such as the Polysomnograph (PSG), that can be used to measure and diagnose sleep apnea with more accurate physiological information than those artificial methods and have already been used to acquire physiological signals during sleep [2,3]. However, they have many limitations, such as complex testing methods, requiring professionals to operate in specific sleeping test rooms, and being complex to wear. Furthermore, the high medical costs of these facilities also cause an economic burden on patients. Therefore, there is an urgent need for the development of portable/wearable hardware and machine learning methods that could be employed for diagnosis of the screening and diagnosis of sleep apneas [4]. Compared to PSG, some portable sleep monitor devices have many advantages, such as low cost and convenience, but the data are much less accurate than those of PSG. As a result, there is considerable room for improvement in portable sleep monitor devices.

The use of portable monitoring devices to evaluate initial practice guidelines for SAS was described by the American Association of Sleep Medicine (AASM) in 1994 [5,6]. In an attempt to lower the cost and complexity of the PSG-based methods, many studies have been done to create new instruments and techniques for screening SAS [7,8]. Various strategies have been developed, and one of the most commonly recommended methods for adults and children is PPG-based screening for SAS [9,10,11].

So far, wearable devices with built-in photoplethysmography (PPG) sensors have become increasingly useful for detecting the physiological characteristics of diseases. PPG is an optical approach that measures blood flow characteristics in the microvascular tissue bed to monitor heartbeats [12]. It uses the light intensity difference to determine physiological parameters, such as heart rate and blood oxygen saturation level, by monitoring light intensities for light passing through the tissue or being reflected by the tissue [13,14]. However, due to numerous environmental noises (optical and electrical) and motion artifacts, the recorded signal may be distorted, resulting in measurement inaccuracies [15,16,17]. Hayano et al. presented a SAS detection system based on wearable watches [18], and Wu et al. developed a SAS screening method based on wearable bracelets [19]. Selvakumar et al. proposed a low-cost Cortex-M4 microcontroller to obtain the photoplethysmography signal and use of an incremental merge segmentation algorithm to measure the respiration rate [20]. On the other hand, measurement errors caused by motion artifacts usually occur when the user moves freely, and patients are required to be in stable condition for tests, which usually means lying in a bed. Therefore, the device for monitoring sleep should be specifically designed to achieve stable measurement. 

According to research, machine learning has been shown to be feasible for SAS diagnosis in a few experiments. With an 83.4% test accuracy, Margot et al. employed a least squares support vector machine classifier to classify the PPG signal and applied it to the PSG data from 102 participants suspected of having sleep apnea-hypopnea syndrome [21]. Debangshu et al. proposed a convolutional neural network-based deep learning framework and employed the single-lead electrocardiogram (ECG) data in PSG to detect SAS automatically with a test accuracy of 98.91% [22]. Lazazzera et al. used a threefold cross-validation method to detect sleep apnea and hypopnea, combining PPG and SpO2 signal fragments, which were used to detect sleep apnea and hypopnea, respectively, by envelope and adaptive threshold estimation and obtained an accuracy of 75.1% in the detection of apnea and hypopnea, and 92.6% in the classification of sleep-disordered breathing by SVM in 2021 [23]. Wei et al. proposed a deep convolutional network MS-net consisting of a multiscale block and shadow module, which used a PPG signal to detect sleep apnea with an accuracy of 87% for the F1-score in 2023 [24]. Yin et al. validated and improved their prediction accuracy through K-fold cross-validation and transfer learning that optimized the detection of sleep apnea in COPD (chronic obstructive pulmonary disease) [25]. Massie et al. used an ensemble of tree classifiers to predict obstructive sleep apnea and evaluated the classifier performance using the leave-one-out cross-validation, which achieved a sensitivity of 81%, a specificity of 99% and an accuracy of 90% in positive prediction in the apnea–hypopnea index [26]. However, most of the approaches still utilized big facilities for testing and were designed for advanced SAS diagnosis rather than feasible devices to screen sleeping-related disorders, limiting their widespread use for large populations. Furthermore, despite their good accuracy, most current diagnostic algorithms for PPG are still in the stage of theoretical study. It is difficult to adapt it to the actual detection environment due to its high complexity, time and resource consumption.

In this work, a wearable wireless sleep monitoring system in the form of a smart ring is proposed, which uses reflective optical PPG sensors to capture PPG data and blood oxygen saturation. Then, the signal is analyzed by using multiscale entropy and random forest algorithms to diagnose SAS. The diagnosis algorithm is quick and efficient, using lower computational resources. The smart ring is worn on a finger, and the signal is transmitted wirelessly to a mobile phone or a PC for real-time analysis, minimizing the complexity of polysomnography and providing a home-based wearable solution for apnea patients.

## 2. Wearable Wireless System Design and Fabrication

**Figure 1:**
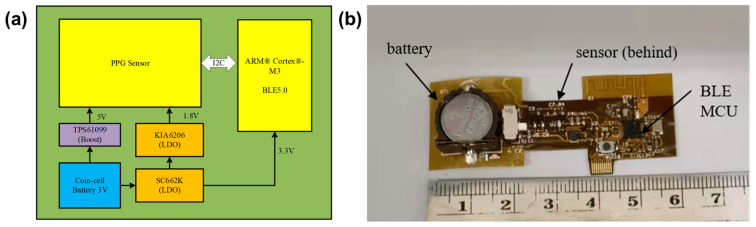
Circuit architecture of the smart ring for the apnea diagnosis (**a**), Photo of the fabricated FPC circuit board (**b**).

**Figure 2:**
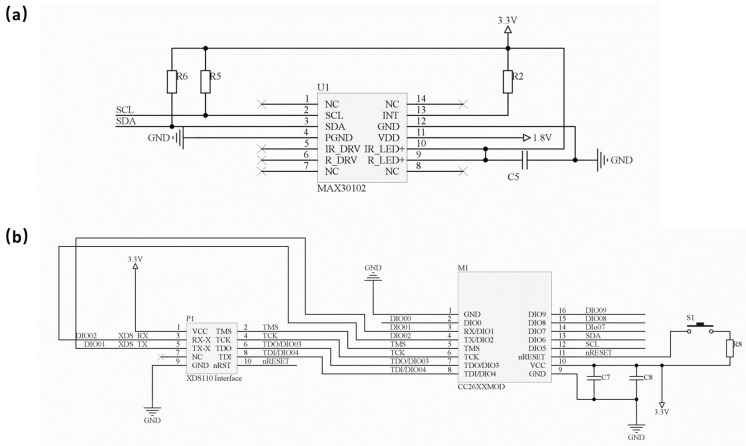
The photoelectric sensor module circuit (**a**); The BLE and MCU module circuit (**b**).

The circuit architecture and fabricated hardware of the wearable apnea diagnostic device are shown in Figure 1a,b, respectively. The flexible device consists of a photoelectric pulse wave sensor module (MAX30102, Analog Devices), a Bluetooth transmission module (CC2640R2F, TI Instruments) and a power supply module. The electronic circuit of the sensor and Bluetooth module are shown in Figure 2a,b.

When a patient wears this device, the physiological characteristic parameters collected by the photoelectric pulse wave sensor module are transmitted to a personal computer (PC) via the Bluetooth Low Energy (BLE) 5.1 protocol. Then, the diagnosis by a specific algorithm developed for this work and the comprehensive diagnosis by the PPG algorithm and SpO_2_ algorithm are carried out, from which an apnea-hypopnea index (AHI) is calculated for classifying the patient’s sleeping breathing situation according to severity.

**Figure 3:**
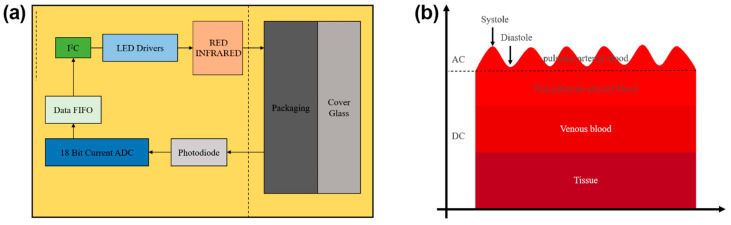
The system block diagram of the sensor (**a**); The measurement principle of a typical PPG signal (**b**).

The photoelectric pulse wave sensor module consists of two light-emitting diodes (LED), red light and infrared light, a photodiode, and an AD converter module. The working principle of the PPG sensor is shown in Figure 3a,b. When the chip MAX30102 chooses the SpO_2_/HR mode, the red light and infrared light alternatively irradiate, controlled by the driver. To measure the red light and infrared light better, we must have a reasonable control of the current intensity and frequency of the LED light, according to need, by programming the driver. A photodiode is used to receive the reflected lights and convert the light signals into electrical signals. The PPG signal is acquired via a high-precision ADC, and the original analog signal is converted into a digital signal.

**Figure 4:**
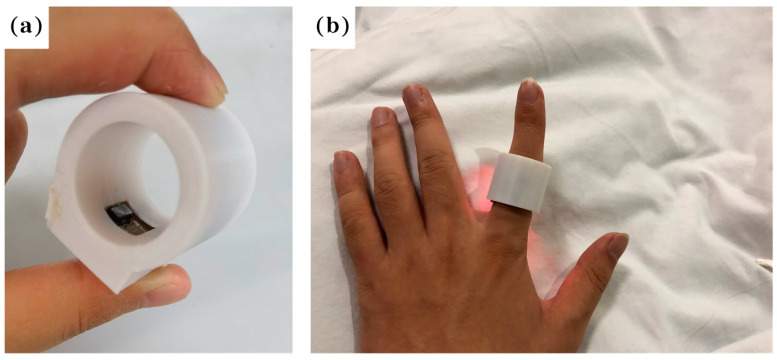
The photo of the fabricated smart ring (**a**); human hand with the smart ring (**b**).

All the electronic components and chips are assembled on a flexible printed circuit board (FPC), as shown in Figure 1b. The FPC board can be bent and folded without damaging the electronic components, and can be placed well within the ring shell, which is 3D printed, as shown in Figure 4a. 

The FPC board with the assembled components and chips is placed on the inner side of the ring to form a smart ring that is used to monitor the sleeping breathing situation when sleeping, as shown in Figure 4b. A mobile phone is used to receive the PPG signal to show the sleeping situation. 

To minimize power consumption, we chose CC2640R2F as the Microcontroller Unit (MCU) with Bluetooth Low Energy (BLE) 5.1 function. The operating current and power consumption of these two components are about 9.1 mA and 30.1 mW, respectively. Those for the MAX30102 device are about 1.2 mA and 6.1 mW, while those for all other parts of the smart ring are about 2.1 mA and 6.0 mW. The total operating current and power consumption of the smart ring are about 12.8 mA and 43.4 mW during the short period of sensing and transmission. A 3 V coin cell battery is used to power the smart ring with a power capacity of 120 mAh, which allows continuous operation of the sleep monitoring device for about 8.2 h, sufficient for the wireless sleeping monitoring application. 

**Figure 5:**
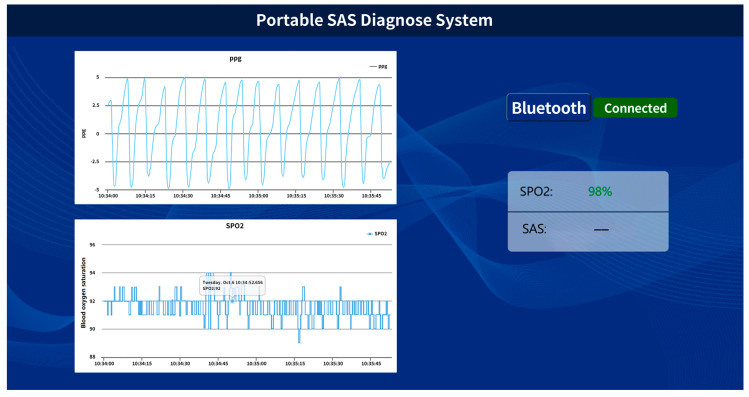
App graphics user interface developed to receive the PPG signal from the smart ring during sleep.

The graphics user interface of the mobile phone for this application is shown in Figure 5, clearly showing the complete cyclic process of users’ PPG signal and SpO_2_ situation during sleep. 

## 3. Diagnostic Algorithm

**Figure 6:**
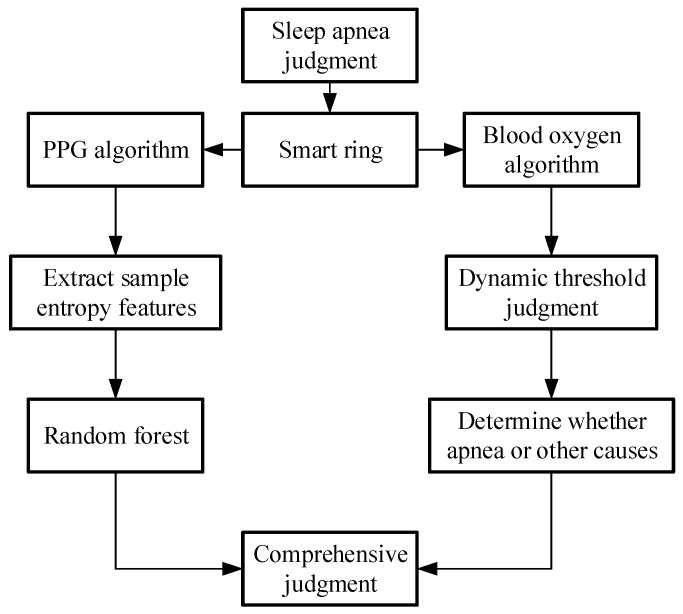
Flow chart of the comprehensive judgement algorithm for apnea.

In this paper, we proposed a comprehensive algorithm, related to the diagnostic result of both the PPG algorithm and the SpO_2_ algorithm. This estimates the number of respirations pause of the patients at a specific time, with an AHI that classifies the patients according to the degree of severity of the situation. The flowchart of the diagnostic algorithm for sleep apnea is shown in Figure 6.

### 3.1. Blood Oxygen Analysis Algorithm

The MCU analyzes the ratio (R) of light intensities of the red and IR lights absorbed by oxygenated hemoglobin (HbO_2_) and deoxyhemoglobin (Hb) in arterial blood and then calculates blood oxygen saturation level using Equation (1), which is the standard formula for measuring the level of blood oxygen saturation.

(1) $$SpO_{2}=\frac{C_{HbO_{2}}}{C_{HbO_{2}}+C_{Hb}}\times 100$$

Here, $C_{HbO_{2}}$ and $C_{Hb}\;$represent the concentration of HbO_2_ and Hb in the blood, respectively.

**Figure 7:**
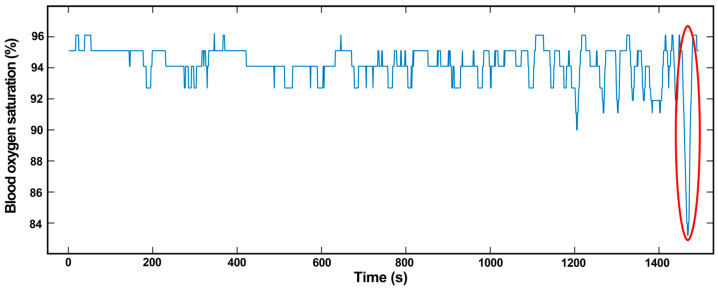
SpO_2_ level variation with time for a wearer is measured by our smart ring. The significantly low SpO_2_ level at the time of 1480 s indicates the sleeping apnea of the wearer.

Apnea can be caused by SAS or other conditions and can be distinguished by the oxygen desaturation index and the oxygen desaturation degree. Oxygen desaturation is a phenomenon that occurs in apnea, which causes a decrease in the SpO_2_ level. Figure 7 shows the SpO_2_ level as a function of time for a wearer during sleep. The SpO_2_ level decreases to less than 90%, which is abnormal.

In general, a decrease in SpO_2_ is not necessarily solely caused by sleep apnea. There are some other factors that may affect the SpO_2_ level, such as a weak PPG signal, whose low frequency can easily be interfered. Hardware may also decrease the SpO_2_ level, such as the loose connection of the electrodes of of the equipment, which will cause mistaken light intensity obtained by equipment to decrease SpO_2_. There are also some contingencies that will cause this issue in detection, such as probe loosening, exercise interference, and weakness perfusion [27]. Therefore, we take the PPG algorithm into consideration with the SpO_2_ algorithm to obtain a better result than that for one index as a diagnostic criterion. 

### 3.2. PPG Analysis Algorithm

For the algorithm used to analyze the PPG signal, we propose an improved method to judge SAS, which combines multivariant multiscale entropy and random forest. This method requires only a small computational resource. Sample entropy refers to judging the complexity of nonlinear finite time series signals by calculating the probability of generating new patterns. Multiscale entropy is to introduce a scale factor on the basis of sample entropy to estimate the complexity of the signal under different scale factors [28]. The PPG data process flow is as follows:

(1) PPG data preprocessing
  This paper proposes PPG data preprocessing method includes three parts, median filter, lowpass filter and signal cutting.
  The PPG signal is influenced by noise from the environment and acquisition equipment during collection, which results in real waveform changes. Therefore, it is necessary to preprocess PPG data, which can avoid the subsequent influence of the error due to noise. PPG signal noise includes the following three types.
  1) Power-line interference:
  Power-line interference, caused by power systems, which is one of the common interferences in ECG signals, is usually removed by digital signal processing.
  2) Electromyography (EMG) interference:
  High-frequency noise interference of the PPG signal is mainly caused by EMG interference. If a person’s muscle or skin has poor contact with the sensor, EMG noise appears, which causes irregular burr in PPG signals that influence signal quality. A lowpass filter is a good way to remove it.
  3) Baseline drift:

Baseline drift of the PPG signal is mainly caused by the electrode, human body and acquisition equipment, which can be classified as low-frequency noise. Superimposed baseline drift will make the PPG signal fluctuation become large, which could result in a subsequent diagnosis. We propose a fast algorithm that uses a median filter with a sliding window length of 2.5N to obtain the baseline drift signal from the original signal first; here, N is the sampling frequency. Then, the filtered signal (baseline drift signal) is subtracted from the original signal, and the signal without baseline drift is obtained. Therefore, we can eliminate baseline drift and reduce the feature loss of signal as much as possible.

After the removal of baseline drift, a specific method of a median filter with a sliding window length of 2N was proposed in this paper to filter out the noise. At first, we define a dispersed signal sequence Y(t) (t>N), assume that at a certain time, signal samples are Y(t−N),……,Y(t−1),Y(t),…..,Y(t+N−1), then we need to sort it according to their numerical values. The median value of sorted Y(t−N),……,Y(t−1),Y(t),…..,Y(t+N−1) is output signal by alternating Y(t−1). The specific formula is shown in Equation (2)

(2) Y(t−1)=Med{Y(t−N),……,Y(t−1),……,Y(t−N+1)}

**Figure 8:**
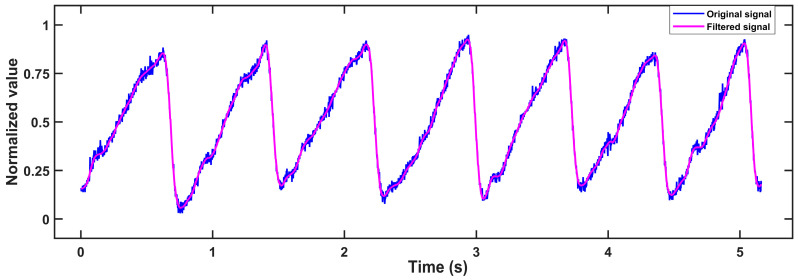
Original signal (blue) and filtered signal (purple).

Then, the signal is passed to a 10-order IIR lowpass filter with a cut-off frequency of 4.8 Hz to inhibit PPG signal noise. Its stopband attenuation is −60 dB, and its passband ripple is 1 dB. Because of the delay phenomenon in the IIR filter, in order to display the filtering effect better, we introduce delay compensation which is to eliminate the first M/2 points in the original signal. Here M is the number of filter orders. The original signal and filtered signal are shown in Figure 8.

For the convenience of algorithm research, we need to cut the signal into one-minute lengths regardless of how it comes from the databases and testing. In the database, the total length of data is several hours, and the label unit of apnea is one minute, which indicates whether apnea has occurred during this period of time. Therefore, we must set a data length window to cut the PPG signal. In measured data, the length of the data window is an important index that balances all aspects of the data. On the one hand, if the length of the window is too short, the selected data will easily miss a complete cycle of apnea. On the contrary, if the length of the window is too long, the selected data will include multiple cycles of apnea, causing a specific weakening of the PPG signal and higher similarity of the extracted feature combination, which will increase the identification difficulty of the algorithm.

After careful consideration, we proposed a 60 s window length. The first 60 s PPG signal is selected for training, and then, sliding the 60 s window to update the data, the data for the first second of the data sequence is deleted and new data is added at the end, which increases the accuracy of the algorithm. This processed PPG signal will increase fault tolerance in algorithm development.

**Figure 9:**
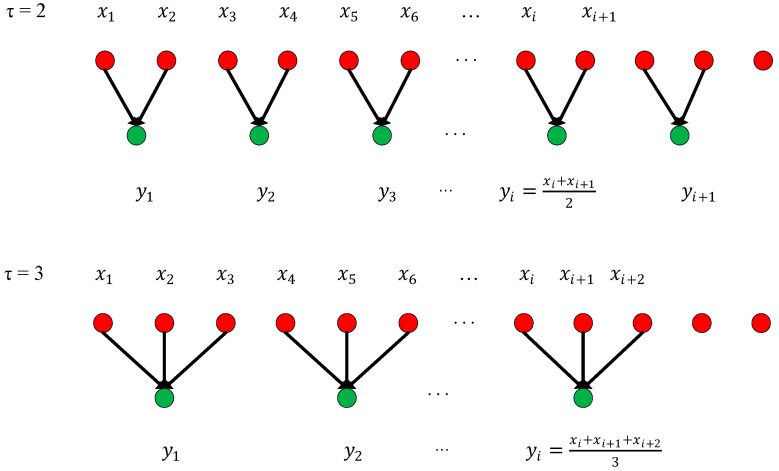
Coarse grain sequences for scale 2 and 3.

**Figure 10:**
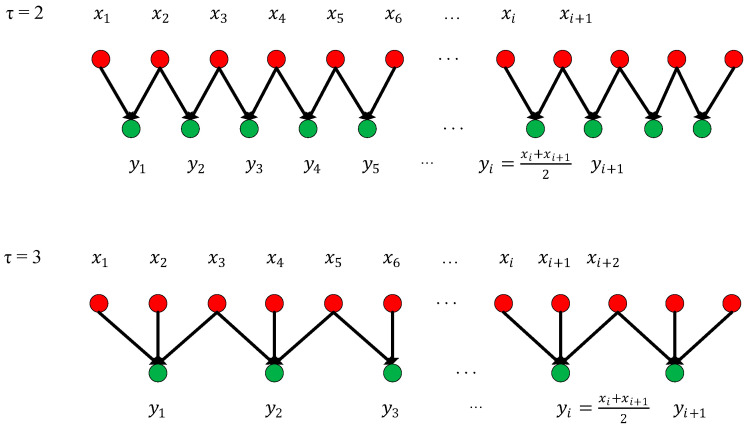
Improved time series coarsening for scales 2 and 3.

(2) multiscale entropy and PRV-feature extraction
  This paper proposes an extraction scheme that combines both multiscale entropy and PRV-feature extraction to extract PPG signal features. This PPG-related algorithm consists of two parts: sequence coarse-graining and sample entropy calculation [29], including three parameters τ, m and r, where τ is the scale factor, m is the embedding dimension, and r is the threshold. The algorithm steps are as follows:
  1) Sequence coarse-grained algorithm:
  In order to improve the accuracy of the multiscale algorithm, an improved coarsening extraction algorithm is proposed. The first step of the traditional coarsening method is to define a discrete one-dimensional time series, $x_{1},x_{2},\ldots \ldots x_{L}$, and then a new time series after coarsening this series is obtained, as shown in Equation (3):
  (3) $$y_{j}^{\tau }=\frac{1}{\tau }\overset{j\tau }{\underset{i=\left(j-1\right)\tau +1}{\sum }}x_{i}$$
  The length of the time series is changed to L/τ after coarsening; when τ = 1, it is the original time series. The method for obtaining the coarse-grained time series with scales 2 and 3 is shown in Figure 9. It can be clearly seen from Figure 9 that the traditional coarsening begins at the first of the time series, and then divides it according to different scale factors. However, the problem is that it will lose some data at the end of the time series because there will appear a situation where the length of one single time series cannot be exactly divisible by the scale factor, which will decrease the accuracy of the multiscale entropy algorithm. Therefore, in order to improve the accuracy of the multiscale algorithm, an improved coarsening extraction algorithm is proposed, which uses a sliding sampling window to sample the original time series and sets the step size to 50% of the window meanwhile. It not only avoids the loss of tail data caused by coarsening at all scales but also decreases errors in later algorithms. The specific process is shown in Figure 10.
  2) Sample entropy calculation:

After coarsening, the sample entropy can be calculated. The specific process is as follows:

i. According to the change of the scale factor τ, a new series is obtained, of which length is $N=\frac{L}{\tau }$, then an m-dimensional vector Y(i) could be constructed as expressed by Equation (4).

(4) Y(i)=[y(i),y(i+1),…,y(i+m−1)]     ( i=1,2,…,N−m+1)

ii. Define d(Y(i),Y(j)) as the maximum absolute value obtained by subtracting two vectors X(i) and X(j) on the scale τ. The formula is shown in Equation (5), where k=0,1,…,m−1; i,j=1,2,…,N−m+1; i≠j.

(5) d(Y(i),Y(j))=max( abs(y(i+k)−y(j+k)))

iii. After setting a threshold r, for each value of index i, calculate the distance between Y(i) and the rest of the vector Y(j) based on Equation (6), where i,j=1,2,…,N−m+1 and i≠j.

(6) $$Cmr=\frac{\left\{d\left(Y\left(i\right),Y\left(j\right)\right)\leq r,j\neq i\right\}}{N}$$

iv. Calculate the average value of Cmr using Equation (7).

(7) MeanCmr=average(Cmr)

v. Let m = m + 1, repeat 2, 3, and 4 to obtain MeanCmr1, and obtain the multiscale entropy value MSE(τ) at scale τ as expressed by Equation (8).

(8) $$MSE\left(\tau \right)=-\ln\frac{\mathrm{average}\left(\mathrm{Cmr}1\right)}{\mathrm{average}\left(\mathrm{Cmr}\right)}$$

Then we can employ the multiscale dividing PPG signal and calculate sample entropy at each scale according to the above process. Each sample entropy includes information on the PPG signal, whether large scale or small, which reflects the complexity of the PPG signals, at their scale size. In this paper, the sample entropy with a scale factor from 1 to 15 is obtained and used as the data source for the following feature extraction.

(3) Feature extraction

After extracting the multiscale entropy features, it is also necessary to extract the Heart Rate Variability (PRV) parameter features based on the PPG signal. PPG-based feature parameters are mainly composed of time-based features and their derived features, such as the mean of peak-to-peak periods and the standard deviation of peak-to-peak periods. Therefore, it is particularly important to locate the peak points first before selecting extraction parameters [30].

As mentioned above, the PPG signal has the characteristics of weak and possible interference. Therefore, a strong anti-interference ability is required for the corresponding peak point detection algorithm, thus reducing the false detection rate and missed detection rate of the peak point. The peak point detection algorithm proposed in this paper is to perform binary wavelet transform on the digital signal. If the intersection points of the rising and falling edges of a signal are the singular point, it is the modulus maximum of the smaller scale. Its location is the zero intersection point of positive and negative maximum pairs of wavelets transform at scale $s=2^{j}$, which is a maximum pair of waveform peaks. The specific detection process is as follows:

1) To obtain the $W_{2^{3}}f\left(n\right)$ value, the Mallat algorithm is used to decompose the input PPG signal at $s=2^{3}$.
2) After detecting the extreme point, diagnose whether the curve between the two extreme points is monotonic. If not, the extremum point is an orphan that needs to be removed. If matching, the positive and negative value pair is retained;
3) Retain the extreme value pair of the maximum positive value $>th_{p}$ and the maximum negative value $<th_{n}$;
4) Detect the peak point between positive and negative value pairs that meet the above conditions, which is the zero-cross point;
5) In order to reduce the false detection rate, the extreme point within 200 ms after the peak point is ignored, which corresponds better to the heart rate characteristics of normal people;
6) In order to reduce the missed detection rate, calculate the average time of the peak-peak cycle at first, then find the segment that has not been detected after 1.5 times the average time of the wave and then re-detect it after changing the threshold to $\frac{4}{5}*th_{p}$ and $\frac{4}{5}*th_{n}$. Finally, restore the threshold after completion.

**Figure 11:**
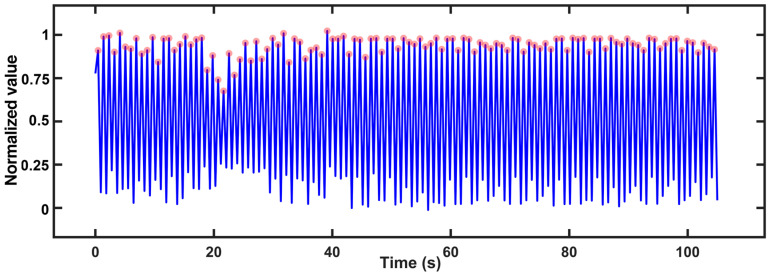
Actual detection effect of peak point detection.

The actual detection effect is shown in Figure 11. The constructed features are listed in Table 1.

Finally, we must have feature selection and evaluation functions; in this research, we select embedded feature selection and a tree model corresponding to the embedded feature to evaluate the importance of the feature and mark it. Then keep the higher important and more efficient feature to form feature engineering by threshold. This proposal not only has better classification ability but also better stability.

## 4. Results and Discussion

### 4.1. Sensing Device Performance Verification

**Figure 12:**
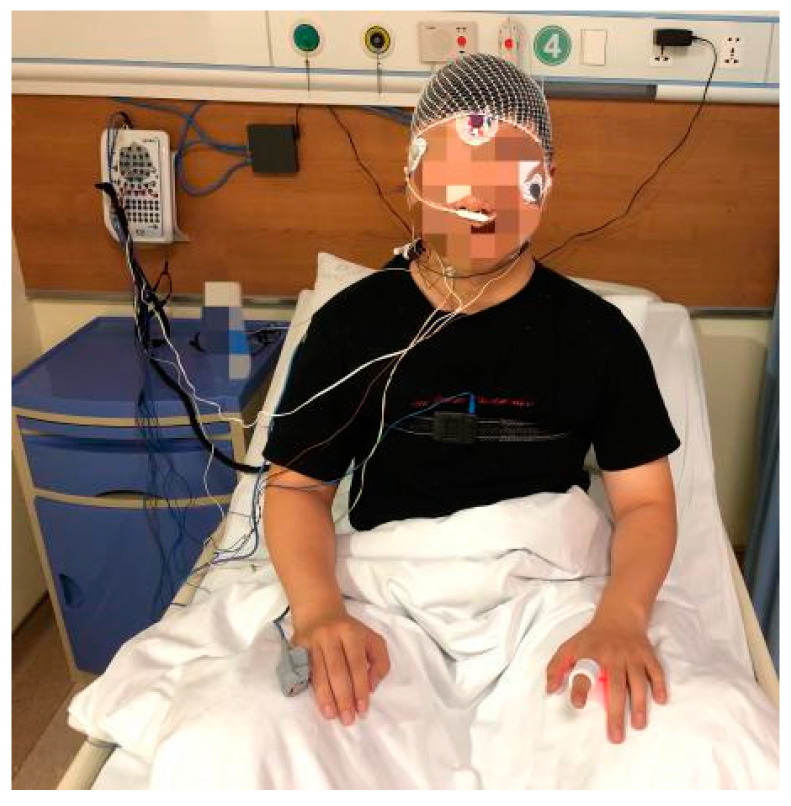
The subject with the smart ring being placed on the right index finger and the professional PSG equipment being placed on the left little finger.

**Figure 13:**
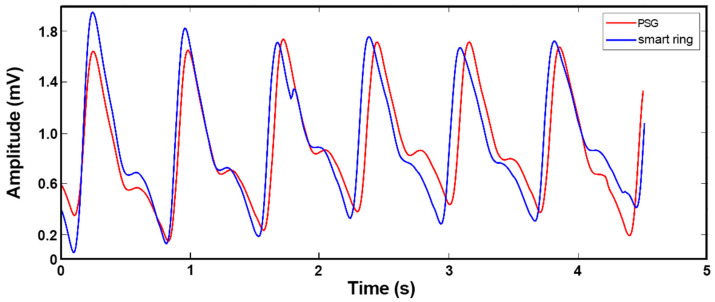
Pulse waves collected by the smart ring (blue) and a professional polysomnograph instrument (red), respectively.

We compared the data from our device with those measured by the commercial Compumedics Grael PSG 45 system, which is a standard instrument for hospitals, in order to verify the performance of our sleep monitoring system. The schematic diagram for clinical data collection is shown in Figure 12. After the Compumedics Grael PSG 45 and wearable smart ring were stabilized, the volunteers wore them while sleeping for a night in a quiet and independent room, and the PPG measurement results were recorded and displayed as graphs simultaneously. Figure 13 shows the pulse waves collected by the smart ring and the Grael PSG 45 sleep monitoring system, respectively. It can be seen that the PPG waveforms obtained from the medical instrument and the smart ring are similar in principle, with some differences in a few features.

**Figure 14:**
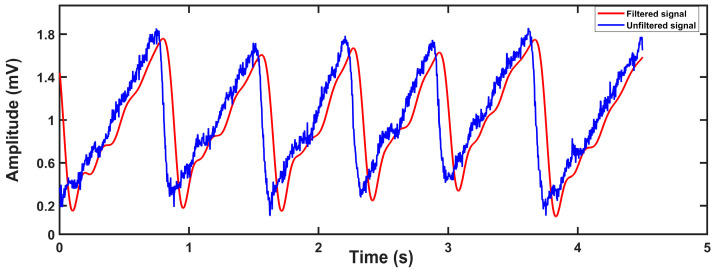
Filtered effect of digital IIR filter.

It should be noted that there are serval parameters that influenced the measurements. Because we used a photoelectric sensor, the main parameter that influenced our measurement results was light. However, there are many environmental factors that can cause changes in light, such as the degree of fitting of the finger to the sensor, which means that we must choose the appropriate finger size for our device to prevent this from affecting the measurement during the patient’s shaking of the finger in their sleep. On the other hand, we inserted a five-order digital IIR filter with a lowpass of the cut-off frequency of 4.8 Hz and a stopband attenuation of −40 dB in our MCU program, so that a cleaner signal was obtained as shown in Figure 14. Although the problem of signal phase delay may be introduced by IIR filters, our research is not very sensitive to the phase of the signal, and therefore it will not affect the results. The calculation formula of signal-noise ratio (SNR) is shown in Equation (9):

(9) $$SNR=10\log\frac{P_{s}}{P_{n}}$$

where $P_{s}$ is the power of the signal and $P_{n}$ is the power of the noise. The SNR of the unfiltered signal is 61.49 dB, so our digital IIR filter helps us filter out a lot of noise.

### 4.2. Algorithm Verification

**Figure 15:**
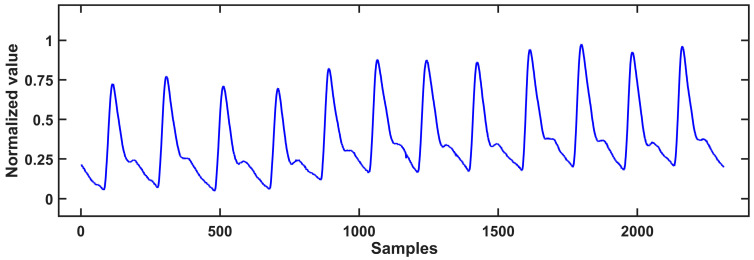
A partial PPG signal diagram of the dataset.

In order to verify the reliability of the algorithms, we performed the algorithm first with public data from the MIT-BIH Polysomnographic Database provided by Boston’s Beth Israel Hospital [31,32]. The database contained records from 16 volunteers aged between 32–56 years old (average age 43 years) and weighing 89–152 kg (average weight 119 kg), including ECG, EEG, pulse pressure, respiratory signals and PPG signals, obtained during sleep. The data recorded every 30 s were accompanied by the relevant sleep stage and apnea marks provided by medical experts. Figure 15 shows a typical partial PPG signal diagram of the dataset.

In terms of machine learning algorithm selection, by comparing better performance supervised algorithms, the random forest classifier was finally selected as the core model of the automatic screening system for apnea diagnosis. Random forest is a supervised ensemble machine-learning algorithm proposed by Breiman [33]. The algorithm generates multiple decision trees at the same time for the input training model; each tree has a weak classifier, and finally, the results of the decision tree are voted on, or bagging methods are used to improve the accuracy of the prediction model [34]. Since each decision tree is independent and then combined together, the random forest algorithm can process overfitting problems better and is able to be processed in parallel with high classification accuracy and fast training speed. It is currently the most researched and used machine learning algorithm in academia and industry. Improved multivariant multiscale entropy was used to extract sample entropy feature vectors of different scales from the PPG signal that were divided into d1, d2, …, d8, which are the sample entropy values obtained after the PPG signal calculated by the sample entropy at different scales. Then, the multiscale sample entropy feature vector was used as the input of the random forest algorithm to analyze the PPG signal.

The training and testing process were run on a computer (CPU: Intel i7 10700 2.9GHz; RAM: 16 GB), and the best model was obtained after repeatedly adjusting parameters. We compared it with a Support Vector Machine (SVM), K-NearestNeighbor (KNN), and XGboost in terms of accuracy (%), sensitivity (%), and specificity (%) at the same setting. The sensitivity reflects the ability of the disease to be identified, as shown in Equation (10).

(10) $$Se=\frac{TP}{TP+FN}$$

The specificity reflects the performance of normal samples not to be misjudged, as shown in Equation (11).

(11) $$Sp=\frac{TN}{TN+FP}$$

The accuracy represents the performance of this system to make correct judgments, as shown in Equation (12):

(12) $$Acc=\frac{TP+TN}{TP+FP+TN+FN}$$

where *TP* is true positives, *TN* is true negatives, *FP* is false positives, and *FN* is false negatives. The comparison results are summarized in Table 2. Among the four classification algorithms, the developed random forest algorithm achieved a specificity of 91.8%, a sensitivity of 89.93%, and an accuracy of 93.88%, much better than those of the other three classification algorithms, as shown in Table 2. It is also superior to other algorithms in terms of running speed. It can be seen that the PPG signal analysis method based on multivariant multiscale entropy and random forest proposed in this paper can effectively identify apnea and has a high recognition accuracy. The SpO_2_ analysis algorithm was used to make additional judgments on whether the decrease of SpO_2_ is caused by probe loosening, exercise interference, or other reasons. If SpO_2_ is decreased due to other reasons, it is determined that the data are incorrect, and an error warning is issued to remind the user to check.

### 4.3. Wearable Diagnostic System Verification

In order to verify the reliability of the wearable apnea diagnosis system, 10 volunteers were selected as subjects to wear both the smart ring and the Grael PSG 45 instrument to collect data throughout the night’s sleep, and then the results were compared with each other. The collected data came from the fittest finger with the largest contact area between the skin and the sensor for both the devices. The ten volunteers were aged between 20–35 years old, some of them were apnea patients, and some were healthy subjects. The pulse duration obtained was the same for both the instruments, and the SpO_2_ level was similar to each other with a relative difference of SpO_2_ within ±2%, which meets the requirements for sleep monitoring. The specific information is shown in Table 3. The volunteers wore the Grael PSG 45 monitoring system for a night in a quiet and independent room. After confirming that the sensors were well connected and the devices were in a normal working state, the volunteers were instructed to sleep when collecting and storing data until the subjects awakened. Then the same procedure was performed with the smart ring system.

For the volunteers’ sleep status diagnosed by the smart ring system, we compared the results with the judgements provided by medical experts. The comparison results are summarized in Table 4. For the ten subjects: the number of apneas of the first subject was judged to be 50 times by our wearable smart ring system, while it was judged to be 56 times by the PSG monitoring expert. The diagnosis accuracy of the system was 86.73%, the specificity was 85.42%, and the sensitivity was 87.42%, respectively. The second volunteer’s apnea times were 17, judged by the smart ring system, while that was judged to be 10 times by the PSG monitoring expert. The system’s accuracy rate was 87.25%, specificity was 90.63%, and sensitivity was 76.34%. The number of apneas in the third subject was 64, according to the smart ring system, and 68 judged by the PSG monitoring expert. The accuracy of our device was 82.25%, the specificity was 65.64%, and the sensitivity was 87.42%. The fourth subject was diagnosed as having apnea 12 times by the smart ring and 15 times by the PSG monitoring expert. Our device’s accuracy rate was 86.34%, specificity was 89.65%, and sensitivity was 79.35%.

The results of the smart ring system show that the accuracy, specificity and sensitivity of our device are good for different volunteers, and the final detection results are more accurate, which meets the basic standards of clinical apnea screening. It should be noted that the wearable smart ring costs less than USD 22; the precise cost of device components and FPCB are listed in Table 5. By contrast, a standard polysomnography machine could cost hundreds of thousands of dollars. The smart ring can be worn on the finger, and the tests can be performed at home, which is a significant improvement over professional polysomnography instrument-based testing. These could make the portable diagnosis of SAS possible at an early stage.

## 5. Conclusions

A wearable, compact, low-cost, wireless apnea diagnosis device was proposed and developed in this paper, and its feasibility was investigated in order to solve the problems of complicated and expensive operation procedures for the clinical detection of sleep apnea, which affects subjects’ sleep comfort and wellbeing.

A ring shape of the apnea diagnostic device can more conveniently monitor sleep by wearing it on one’s finger without disturbing patients’ sleep. Physiological data can be transferred wirelessly to a mobile phone or computer for examination. The gadget system’s software generates a diagnostic model based on pulse and blood oxygen signals. It is not only favorable to early detection of apnea, but it also improves the diagnostic rate and reduces the sudden mortality rate. It also helps to minimize medical costs, according to its strong prediction capacity and generalization ability. It is of significant scientific and practical use to apply this technology in the diagnosis of apnea.

Although this study employing the smart ring and polysomnography instrument model was undertaken for comparative analysis, the number of clinical tests was modest, and the amount of data was small compared to the complexity of PSG monitoring. As a result, in the next stage of collecting clinical data with the smart ring device, the system hardware and software in the application must be improved and optimized on a regular basis. We have applied the model to test the condition of sleep apnea, but we have not yet defined the precise forms of sleep apnea in the patient, which will require more complex classification in the future.

## Figures and Tables

**Table 1:** The parameter definition extracted, based on PRV analysis.

Parameter	Definition of the Parameter
PR	Pulse beats per minute, reflecting breathing rate
$\bar{\mathrm{PR}}$	The mean value of the peak-to-peak period, indicating the average level of PRV
SDNN_PP_	Standard deviation of peak-to-peak period
PP_median_	The median periods of the peak-to-peak
NN50	The number of times the difference between two adjacent peak-to-peak intervals exceeds 50ms, indicating the beat-to-beat variability of the pulsatile cycle
PNN50	The number of times the difference between two adjacent peak-to-peak periods exceeds 50ms as a percentage of the total number of peak-to-peak periods
SDNN_AA_	Standard deviation of peak-to-peak amplitude
$\bar{\mathrm{AA}}$	The average of peak-to-peak amplitude
AA_median_	The median of peak-to-peak amplitude

**Table 2:** The performance of sleep apnea judgment under different models.

Models	Accuracy (%)	Specificity (%)	Sensitivity (%)	Running Time (s)
Random Forests	91.80	89.93	93.88	0.21
SVM	88.28	91.69	83.94	2.10
KNN	85.06	86.11	83.72	0.36
XGboost	82.05	84.91	78.42	0.54

**Table 3:** Basic information on 10 subjects.

No.	Gender	Age (Years)	Height (cm)	Weight (kg)	Blood Oxygen Saturation			NO of SAS	
					PSG	Our System	Relative Error (%)	Determined by the Hospital	Determined by Our System
1	Male	23	176	60	99	99	0	56	50
2	Male	32	170	78	98	97	1.02	10	17
3	Male	28	169	68	98	98	0	11	5
4	Male	29	175	80	97	98	1.03	15	12
5	Female	48	163	63	97	97	0	81	80
6	Female	52	161	58	98	98	0	79	77
7	Male	47	173	81	97	98	1.03	68	64
8	Male	54	169	72	98	96	2.08	129	116
9	Female	23	159	55	99	98	1.01	12	7
10	Female	36	154	61	97	96	1.03	85	73
average					97.8	97.5	0.31		

**Table 4:** Comparison of results obtained by system and medical experts.

Subject No.	Accuracy (%)	Specificity (%)	Sensitivity (%)
1	86.73	85.42	87.42
2	87.25	90.63	76.34
3	82.25	65.64	87.42
4	86.34	89.65	79.35
5	89.39	83.47	88.01
6	84.98	83.91	87.11
7	81.87	76.33	86.91
8	85.97	89.25	81.21
9	87.03	81.96	85.23
10	88.08	89.52	80.82

**Table 5:** Cost of device components (basic elements include resistors and capacitors, etc.).

Component Names	Number	Cost ($)
MAX30102	1	1.78
CC2640R2F	1	2.24
TPS61099	1	0.63
KIA6206	1	0.71
SC662K	1	0.18
FPCB	1	14.78
Basic components	/	0.71
3D print shell	1	1.44

## Data Availability

Data are available upon request by contacting the corresponding author.
